# An Account of the Jail Fever, or Typhus Carcerum; as It Appeared at Carlisle in the Year 1781

**Published:** 1782

**Authors:** 


VI. *An account of the Jail Fever, or Typhus Carcerum; as it appeared at Carlisle in the year* 1781. *By* John Heysham, M. D. 8vo. Cadell, *London*, 1782. 59 pages. 1s. 6d.

ALTHO' this fever arose neither in a Jail nor an Hospital, yet it so exactly resembled the Jail fever, both in the symptoms, causes, and method of cure, that our author has thought it right to describe it under that name.

The work is divided into five sections. In the first section the author treats of the symptoms and history of this fever.

It first appeared, we are told, about the latter end of March, in a house, which contained several very poor families, crowded into small, close apartments, the confined air of which was rendered still more noxious by the filth of the inhabitants.

One of the persons affected with the fever in this house was a weaver, who, on his recovery, went to his usual employment at a large workshop, where he communicated the disorder to his fellow weavers, and from thence the fever spread through every part of the town, but was confined almost entirely to the common and lower ranks of people, and raged chiefly amongst those who lived lived in narrow, close, confined lanes, and in small, crowded apartments.

It attacked adults more frequently than young persons. Our author saw no children under three years of age who were affected with it. He observed likewise, that married persons were more subject to it than the single. It was in a great measure confined to Carlisle, for altho' the infection was carried to two of the neighbouring villages, it did not spread much in either of them.

Dr. Heysham has endeavoured to ascertain the number of persons who were seized with this fever, and from the best information he has been able to procure, it appears that from March 1781 to January 1782, near six hundred, or about one in eleven or twelve of all the inhabitants of Carlisle, had been affected with it, and that of this number about one in ten died.

After these remarks, and some others on the state of the air, we are presented with a history of the disease, which seems to be related with great accuracy. It corresponds with the accounts given of this fever by Pringle and other writers.

Being persuaded of the general truth of the doctrine of critical days, our author looked for them in the present case with some attention, but as far as he could observe this fever had no stated or certain duration. An early or a late, a falutary or a fatal termination of this complaint, entirely depended upon the conftitution of the patient, and the fpeedy application of proper remedies.

In the fecond fection Dr. Heyfham treats of the prognoftics. He obferves that petechiæ, which fometimes occurred in this fever, did not portend imminent danger; and that a bleeding of the nofe, unlefs accompanied with other dangerous circumftances, did not appear to be an alarming fymptom. Neither of thefe fymptoms, however, we are told, happened in patients who began to take bark and wine early in the difeafe.

In the third fection the author fpeaks of the occafional caufe of this fever, which he very properly afcribes to human effluvia, uninfluenced by any particular ftate of the atmofphere. In the following fection he enumerates the predifpofing caufes. Thefe are fuch as tend to weaken and debilitate the body.

The fifth and laft fection is allotted to the cure. The mode of treatment adopted by our author feems to have been very judicious. His firft care was to remove, if poffible, every caufe which could tend to aggravate the diforder. For this purpofe he prefcribed clean linen and frefh air.

He

He was likewiſe careful to guard againſt every thing that might occaſion fear and anxiety in his patients. He mentions ſeveral cuſtoms prevalent at Carliſle, which, notwithſtanding his attention to this point, tended much to alarm and terrify the ſick. One of theſe is the *death-bell*, which tolls upon the death of every inhabitant. This cuſtom is pretty general, we believe, throughout England. Another is the public cryer's ringing his bell and proclaiming in every ſtreet, in a loud tone of voice, the hour of the deceaſed's funeral, inviting the friends and neighbours to attend. Laſtly, funeral pſalms are ſung by the attendants as they are conducting the corpſe through the public ſtreets to the church-yard, for interment. The ill effects of theſe cuſtoms, in a caſe of epidemical ſickneſs like the one here ſpoken of, are very properly pointed out by our author.

At firſt, when there were ſymptoms of coſtiveneſs he gave ſmall doſes of tartar emetic; but, having obſerved in ſeveral caſes where it excited vomiting, that all the ſymptoms of the diſeaſe were conſiderably aggravated, he altogether laid it aſide. His chief reliance was on a liberal uſe of bark and Port wine. Theſe he adminiſtered early in the diſeaſe. The bark, if the patient's ſtomach would bear it, he preſcribed in in ſubſtance, in doſes of two ſcruples every ſecond or third hour in a draught of red wine, with the addition of ten grains of *pulv. rad. ſerpent. virg.* if not, he gave it in decoction.

According to the age, ſex, or conſtitution of the patient, and the urgency of the ſymptoms, different quantities of wine were adminiſtered. To adults he uſually ordered from one to two bottles and a half in the ſpace of 24 hours, and he never ſaw any bad effects from an exceſs, though he has ſometimes perceived evident ones from a too ſparing uſe of this grateful cordial.

Bliſters ſeemed to have little or no effect upon the general ſyſtem; but, when applied behind the ſhoulders, were evidently of uſe in abating the pain of the head.

The pediluvium, on account of the fatigue that attended it, in general ſerved only to debilitate the patient; but fomentations of the feet and legs with flannel wrung out of hot water were of uſe.

When a diarrhœa occurred, our author loſt no time in endeavouring to check it; firſt by combining opiates with the bark, and if theſe failed, by diſcontinuing the latter and increaſing the quantity of wine, with the addition of a cordial aſtringent, till the purging was ſtopped.

In

In cafes of extreme debility, though not attended with a diarrhœa, he frequently adminiftered opium (as a more inftantaneous and diffufive ftimulant than either wine or bark) with confiderable advantage.

A few patients were let blood, fome in confequence of advice, and fome of their own accord; but of thefe, we are told, fcarcely one efcaped.

